# Identification of Secretory Proteins in* Mycobacterium tuberculosis* Using Pseudo Amino Acid Composition

**DOI:** 10.1155/2016/5413903

**Published:** 2016-08-11

**Authors:** Huan Yang, Hua Tang, Xin-Xin Chen, Chang-Jian Zhang, Pan-Pan Zhu, Hui Ding, Wei Chen, Hao Lin

**Affiliations:** ^1^Key Laboratory for Neuro-Information of Ministry of Education, School of Life Science and Technology, Center for Informational Biology, University of Electronic Science and Technology of China, Chengdu 610054, China; ^2^Department of Pathophysiology, Southwest Medical University, Luzhou 646000, China; ^3^Key Laboratory of Network Oriented Intelligent Computation, Harbin Institute of Technology Shenzhen Graduate School, Shenzhen, Guangdong 518055, China; ^4^Department of Physics, School of Sciences, Center for Genomics and Computational Biology, North China University of Science and Technology, Tangshan 063000, China

## Abstract

Tuberculosis is killing millions of lives every year and on the blacklist of the most appalling public health problems. Recent findings suggest that secretory protein of* Mycobacterium tuberculosis* may serve the purpose of developing specific vaccines and drugs due to their antigenicity. Responding to global infectious disease, we focused on the identification of secretory proteins in* Mycobacterium tuberculosis*. A novel method called* MycoSec* was designed by incorporating *g*-gap dipeptide compositions into pseudo amino acid composition. Analysis of variance-based technique was applied in the process of feature selection and a total of 374 optimal features were obtained and used for constructing the final predicting model. In the jackknife test,* MycoSec* yielded a good performance with the area under the receiver operating characteristic curve of 0.93, demonstrating that the proposed system is powerful and robust. For user's convenience, the web server MycoSec was established and an obliging manual on how to use it was provided for getting around any trouble unnecessary.

## 1. Introduction

*Mycobacterium tuberculosis* (*M. tuberculosis* or MTB), also known as acid-fast bacilli, is the causative pathogen of the contagious disease tuberculosis (TB). Over the two decades, despite booming development of molecular technologies and diagnostic platforms, TB remains a disastrous global health threat ranking alongside the human immunodeficiency virus (HIV) as the second killer worldwide [1]. There were an estimated 9.6 million new TB cases in 2014, and China is bearing 10 percentage of the global total according to the latest World Health Organization (WTO) report [2]. To reverse the severe situation, constant efforts on developing effective vaccines as well as controlling the spread of MTB are in long term needed. Since delayed or incorrect diagnosis of TB markedly aggravates the epidemic of* M. tuberculosis*, it is urgent to develop method for credible early-stage diagnosis of TB and comprehensive understanding of the intrinsic pathogenesis of* M. tuberculosis*, especially the multidrug-resistant strains of* M. tuberculosis* (MDR-TB).

Recent researches suggest that secretory protein antigens can be used to detect antibodies in infected specimens [3]. And it is a well-established fact that effector proteins are mostly secretory proteins that stimulate infection by manipulating the host response [4]. In line with nonsecretory proteins,* M. tuberculosis* secretory proteins are also assembled in ribosomes but transported to extracellular milieu through certain specific secretion system to attack host cells for survival and reproduction, thus changing the host cell microenvironment and causing grievous tubercular symptoms in infected individuals. Hence, distinguishing secretory proteins from nonsecretory proteins is a matter of grave concern for tracing the real pathogenic factors and developing vaccines or drugs against TB.

Benefited from the next-generation sequencing technology, the amount of protein sequences is exploding with an exponential growth, which is far beyond the capacity of classical biochemical analysis through advances in experimental facilities and molecular biotechnologies. Faced with such an embarrassment, appropriate and fast computational methods exploit a fresh avenue to investigate the properties of* M. tuberculosis* proteins. However, to the best of our knowledge, few computational works focused on secretory proteins in* M. tuberculosis*. Since a majority of secretory proteins have a signal peptide by which they were exported via the signal peptidase pathway, in 2009, Leversen et al. evaluated the performance of nine signal peptide prediction algorithms for identification of mycobacterial signal peptides using sequence data from proteomic methods [5]. Sequential and structural characteristics of secretory proteins also help to address the problem. In 2010, Vizcaíno et al. identified potential secretory proteins from* Mycobacterium tuberculosis* H37Rv by screening its genomes using machine-learning tools [6]. The prediction of subcellular locations of* M. tuberculosis* proteins also provides vital clue for identifying secretory proteins. Recently, Zhu et al. used a support vector machine- (SVM-) based model to predict the subcellular localization of mycobacterium proteins [7]. The success on mycobacterium proteins as mentioned above suggested that bioinformatics approaches are effective strategy for mining useful information and providing insights into both basic research and drug design.

Thus, the current study was devoted to develop a bioinformatics approach to identify secretory proteins in* M. tuberculosis.* The work will describe how to construct a valid benchmark dataset, how to develop an effective mathematical expression to formulate the proteins, and what kinds of machine-learning method and cross-validation tests were used in the prediction model. Finally, based on the proposed method, a web server called* MycoSec* was established.

## 2. Material and Methods

### 2.1. Benchmark Datasets

The original datasets used in this study were extracted from the Universal Protein Resource (UniProt) [8]. To guarantee a good quality of data, proteins in UniProt were collected confidently according to the following criteria: (I) only those from* M. tuberculosis* were considered; (II) only those reviewed and annotated by experts were chosen; (III) sequences with ambiguous residues, such as “*B*,” “*X*,” and “*Z*,” were discarded; (IV) sequences that were inferred from homologous proteins were eliminated; (V) sequences that were fragments of other proteins were excluded; (VI) sequences that have less than 16 amino acids were removed to meet the parameter (*λ*) requirement (see Section 3.1); (VII) sequences with the keyword “secreted” or “secretory vesicle” in the “subcellular location” column were regarded as secretory proteins (positive samples), while sequences without these keywords were considered as nonsecretory proteins (negative samples or control samples). After going through the above processes, a total of 420 samples were reserved, including 63 positives and 357 negatives. To exclude any homologous bias which may cause overestimation problem of final prediction results, we clustered these protein sequences with the CD-HIT program [9] by setting 30%, a rigorous value of this parameter, as the cutoff threshold of sequence identity to remove the redundant part. As a result, the benchmark dataset **S** containing 35 secretory proteins and 266 nonsecretory proteins can be expressed as

(1) $$\begin{array}{c} S=S^{+}\cup S^{-}, \end{array}$$

where **S** ^+^ represented the positive subset, **S** ^−^ represented the negative subset, and the symbol “∪” represents “union” in the set theory. More detailed information of the datasets can be downloaded from http://lin.uestc.edu.cn/server/MycoSec/data.html/.

### 2.2. The Representation of Protein Samples

Translating conventional biological problems into computable mathematical models is usually adopted as an accommodation to the requirement of bioinformatics analysis. A protein sequence with total number of *L* amino acids is usually formulated as

(2) $$\begin{array}{c} P=R_{1}R_{2}R_{3}\cdots R_{L}, \end{array}$$

where **R**_*i*_  (*i* = 1,2, 3,…, *L*) denotes the *i*th amino acid residue in the query protein sample **P**, of which *L* is the length of the protein. Almost all the existing machine-learning methods, regardless of supervised and unsupervised or semisupervised, such as SVM [10–13], Artificial Neural Network (ANN) [14], *K*-Nearest Neighbor (KNN) [15], and ensemble classifiers [16–18], can only handle vectors with the same dimension rather than sequence samples [19]. Meanwhile, data discretization is also required by mainstream feature selection strategies [20–22]. Accordingly, the concept of discrete vector is proposed to realize more general representations of sequence fragments.

The pseudo amino acid composition (PseAAC) is a widely used method for representing protein sequences for its annexation of long-range sequence-order information and the correlation of physicochemical properties between two residues, as well as its balance between representative capability and computational expense. Inspired by PseAAC, we made an improvement by substituting amino acid composition for *g*-gap dipeptide composition. Thus, each protein sequence in our benchmark dataset can be denoted by a 400 + *nλ* dimension vector; that is,

(3) $$\begin{array}{c} P={\left[\;x_{1},x_{2},\ldots ,x_{400},x_{400+1},\ldots ,x_{400+n\lambda }\;\right]}^{T}, \end{array}$$

where the first 400 elements *x*_1_, *x*_2_,…, *x*_400_ are the *g*-gap dipeptide composition and the next *nλ* elements *x*_400+1_, *x*_400+2_,…, *x*_400+*nλ*_ are the first tire to *λ*th tire correlation factors of protein sequence, which are determined on the basis of physiochemical properties. “*T*” is a symbol of transpose operator. *x*_*u*_  (*u* = 1,2,…, 400,…, 400 + *nλ*) can be calculated as given in

(4) xu=fu∑i=1400fi+ω∑j=1nλτj1≤u≤400ωτu∑i=1400fi+ω∑j=1nλτj400+1≤u≤400+nλ,

where *f*_*u*_  (*u* = 1,2,…, 400) is the occurrence frequency of the *u*th dipeptide in **P**, as formulated by

(5) $$\begin{array}{c} f_{u}=\frac{n_{u}^{g}}{\sum_{u=1}^{400}n_{u}^{g}}=\frac{n_{u}^{g}}{L-1-g}. \end{array}$$

*n*_*u*_^*g*^  (*u* = 1,2,…, 400) is how many times the *u*th *g*-gap dipeptide appears in **P**. *ω* is the weight coefficient. *n* is the number of physicochemical properties selected. *λ* is the number of total counted ranks or tires of the correlations along a protein sequence and *τ*_*j*_  (*j* = 1,2,…, *nλ*) is the *j*th tire correlation factor that reflects the sequence-order correlation between all the *j*th most contiguous dipeptides along a protein sequence, as formulated by the following formula:

(6) $$\begin{array}{c} \tau_{1}=\frac{1}{L-1}\overset{L-1}{\underset{i=1}{\sum }}H_{i,i+1}^{1}\\ \tau_{2}=\frac{1}{L-1}\overset{L-1}{\underset{i=1}{\sum }}H_{i,i+1}^{2}\\ \vdots \\ \tau_{n}=\frac{1}{L-1}\overset{L-1}{\underset{i=1}{\sum }}H_{i,i+1}^{n}\\ \tau_{n+1}=\frac{1}{L-2}\overset{L-2}{\underset{i=1}{\sum }}H_{i,i+2}^{1}\\ \tau_{n+2}=\frac{1}{L-2}\overset{L-2}{\underset{i=1}{\sum }}H_{i,i+2}^{2}\\ \vdots \\ \tau_{2n}=\frac{1}{L-2}\overset{L-2}{\underset{i=1}{\sum }}H_{i,i+2}^{n}\\ \vdots \\ \tau_{n\lambda -1}=\frac{1}{L-\lambda }\overset{L-\lambda }{\underset{i=1}{\sum }}H_{i,i+\lambda }^{n-1}\\ \tau_{n\lambda }=\frac{1}{L-\lambda }\overset{L-\lambda }{\underset{i=1}{\sum }}H_{i,i+\lambda }^{n}\\ \;\left(\lambda <L\right), \end{array}$$

where the correlation function **H**_*i*,*i*+*λ*_^*n*^ is calculated by

(7) $$\begin{array}{c} H_{i,i+\lambda }^{n}=h^{n}\left(\;R_{i}\;\right)\cdot h^{n}\left(\;R_{i+\lambda }\;\right), \end{array}$$

where *h* ^*n*^(*R*_*i*_) denotes the constant value of the *n*th kind physicochemical property for **R**_*i*_ and *h* ^*n*^(**R**_*i*+*λ*_) denotes the *n*th physiochemical property value for **R**_*i*+*λ*_. Due to the different scales of values among various properties, the following normalization process is necessary to attain nondimensional data:

(8) hkRi=h0kRi−∑v=120h0kRv/20∑u=120h0kRu−∑v=120h0kRv/202k=1,…,n,

where *h*_0_^*k*^(**R**_*i*_) is the original value of the *k*th kind physicochemical property for **R**_*i*_, **R**_*v*_  (*v* = 1,2,…, 20) stands for the *v*th category amino acid residue, and **R**_*u*_  (*u* = 1,2,…, 20) is defined in the same manner as **R**_*v*_. The normalized values obtained by (8) will have a zero mean value over the 20 kinds of amino acids and will remain unchanged if going through the same conversion procedure again.

It is widely accepted that physicochemical properties of proteins have impacts upon not only a series of biological processes [23], such as protein denaturation and renaturation, cell signaling transduction, and change of solution conductivity, but also maintaining tertiary structures, further underlying certain molecular functions. Among various properties, hydrophilicity and hydrophobicity were chosen here on the basis of a priori knowledge that water is the environment for the survival of all biological molecules and promotes the transportation of molecules by an intrinsic liquidity. In our model, the tire correlation of hydrophilic and hydrophobic amino acids was investigated by tuning the parameter *λ* in order to see the global placement in which amino acids were linked to each other.

### 2.3. Support Vector Machine

SVM is a welcome and powerful machine-learning algorithm which has been successfully used in the realm of bioinformatics for pattern recognition and classification. Established on the theories of Vapnik-Chervonenkis Dimension [24] (VC Dimension) and structural risk minimization, SVM can solve binary nonlinear classification problems among small samples and achieve good generalization ability by mapping the input data into higher dimensional feature space (Hibert space) by kernel function transformation and then determining an optimal hyperplane to separate a given set of labeled data. For multiclass classification tasks, “one-versus-one (OVO)” and “one-versus-rest (OVR)” are generally applied to extend the traditional SVM. A brief description of formulation of SVM is given in [25, 26] and, for more details, please see a monograph [27].

In this work, the toolbox LIBSVM 3.20 was employed to implement the SVM classifier and perform the prediction, which can be freely downloaded from http://www.csie.ntu.edu.tw/~cjlin/libsvm/. The feature vectors of protein samples formulated by (3) were used as inputs of the SVM. Radial basis function (RBF) was adopted here on account of better validity, less deviation, and faster speed in nonlinear training process compared with other kernel functions. For pursuit of the optimal model, the penalty constant *C* and the kernel width parameter *γ* were tuned in an optimization procedure using a grid search method, of which the search spaces for *C* and *γ* are [2^15^, 2^−5^] and [2^−5^, 2^−15^] with steps of 2^−1^ and 2, respectively.

### 2.4. Performance Evaluation

Here we attempted to evaluate the performance of a statistical predictor from two parts: (a) evaluating its generalization ability with an impartial cross-validation method; (b) employing appropriate metrics to measure its success rate.

Independent dataset test, subsampling (or* k*-fold cross-validation) test, and jackknife test are the three widely used validation methods to evaluate the anticipated success rate of a predictor [28]. Among the three methods, jackknife test is deemed the least arbitrary due to its basic idea of leaving out each sample as the testing set iteratively and then finding the mean value of these calculations. However, compared to the other two methods, jackknife test is so time-consuming that it lags the overall computational efficiency. Thus, to balance between correctness and efficiency, a trade-off was made by applying fivefold cross-validation test in the stage of parameter optimization and then switching to jackknife test for evaluation of final model once the optimal model was found.

Here, a set of straightforward methods was provided to assess the prediction quality by using the following three metrics: sensitivity (Sn) which is also known as recall, specificity (Sp), and average accuracy (AA), which are defined as follows:

(9) Sn=TPTP+FN0≤Sn≤1,Sp=TNTN+FP0≤Sp≤1,AA=Sn+Sp20≤Ac≤1.

In (9), TP, TN, FP, and FN are the number of true positives, true negatives, false positives, and false negatives. Sn indicates the ability of correctly judging positive samples, Sp suggests the ability of correctly recognizing negative samples, and Ac averages Sn and Sp.

The Receptor Operating Characteristic curves [29] (ROC curves) were also plotted to describe the performance of models by plotting the Sn (true positive rate (TPR)) against the 1-Sp (false positive rate (FPR)) across the entire range of SVM decision values, under which the area (AUC) can serve as an objective indicator for quality assessments even when the classes are of very different sizes. The value 0.5 of AUC is equivalent to random prediction while 1 represents a perfect one. Accordingly, the larger the AUC is, the more credibility the predictions have.

### 2.5. Feature Selection

Feature selection is very important in view of dimension reduction for pinpointing distinguishing features and then improving generalization ability [30]. In the present study, we performed feature selection by using the Analysis of Variance (ANOVA), which is known as a robust and simple method to test the difference in means between groups, even when the number of observations is uneven in each group. Besides, it is easily generalized to more than two groups without increasing the Type 1 error. According to the basic idea of ANOVA, features can be ranked by their* F*-values calculated as follows:

(10) $$\begin{array}{c} F\left(\;u\;\right)=\frac{S_{B}^{2}\left(u\right)}{S_{W}^{2}\left(u\right)}, \end{array}$$

where **S**_**B**_^2^(*u*) and **S**_**W**_^2^(*u*) denote the sample variance between groups (also called Mean Square Between, MSB) and sample variance within groups (also called Mean Square Within, MSW) separately; the detailed formulae were given in

(11) SB2u=∑i=12mi∑j=1mixui,j/mi−∑i=12∑j=1mixui,j/∑i=12mi2−1,SW2u=∑i=12∑j=1mixui,j−∑j=1mixui,j/mi∑i=12mi−2,

where *x*_*u*_(*i*, *j*) is the value of the *u*th feature of the *j*th sample in the *i*th group defined in (4) and *m*_*i*_ is the sample size of each group (here *m*_1_ = 35 and *m*_2_ = 266). For only two groups were counted in the current study, the degree of freedom (DOF) between and within groups was 1 and (*m*_1_ + *m*_2_ − 2), respectively. Still, it is apparent that a larger value of *F*(*u*) reflects a better discriminative capability of a feature.

Based on the features thus ranked, we used the incremental feature selection [31] (IFS) to determine the optimal number of features as described below. The first feature subset was initialized by a feature with the highest* F-*value, and the next subset was composed when the feature with the second highest* F*-value was added. We repeated this process by adding features sequentially from higher to lower* F*-values until all candidate features were added. Thus, the *N* feature sets thus formed would be composed of *N* ranked features. The *ζ*th feature set can be formulated as

(12) $$\begin{array}{c} S_{\zeta }=\left[\;F_{1},F_{2},\ldots ,F_{\zeta }\;\right]\;\left(1\leq \zeta \leq N\right). \end{array}$$

For each of such *N* feature sets, an SVM prediction model was constructed and examined by the jackknife test on the benchmark data set. Afterwards, we obtained an IFS curve in a 2D Cartesian coordinate system with index *ζ* as the abscissa (or* X*-coordinate) and the AUC as the ordinate (or* Y*-coordinate). The optimal feature set is expressed as

(13) $$\begin{array}{c} S_{\gamma }=\left[\;F_{1},F_{2},\ldots ,F_{\gamma }\;\right]\;\left(1\leq \gamma \leq N\right) \end{array}$$

with which the IFS curve reaches its peak. In other words, in the 2D coordinate system, the AUC value reaches its maximum when *X* = *γ*.

## 3. Results and Discussions

### 3.1. Parameter Optimization

As we can see from (3) to (8), the results of the proposed method depended on three parameters, that is, *λ*, *g*, and *ω*. *λ* represents the tiers counted for the global or long-range sequence-order effect and larger *λ* may contain more global sequence-order information; *g* portrayed the local or short sequential tendencies by measuring the gap between two amino acid residues; and *ω* is the weight factor imposed between local and global effects which is usually within the limits of 0 to 1. To search for the optimal values of the three parameters which can achieve the highest accuracy, we performed a series of experiments according to the following standard:

(14) 0≤g≤9with  step  Δ=1,1≤λ≤15with  step  Δ=1,0≤ω≤1with  step  Δ=0.1.

Hence, a total of 10 × 15 × 11 = 4950 individual combinations (or points in the 3D parameter space) had to queue up to be screened for finding the optimal one, which was actually a routine but tedious process to optimize the model via a 3D grid search [32]. In view of computational efficiency, fivefold cross-validation was firstly employed to cope with the prioritization of parameters, and once the optimal values were determined, the rigorous jackknife test was performed to evaluate the success rates of the feature set according to the four metrics defined in* Performance Evaluation* section. As a result, the largest AUC of 0.845 was obtained when *g* = 9, *λ* = 6, and *ω* = 1.

The dimension of the optimal feature set was 400 + 2 × 6 = 412, which means that 400 9-gap dipeptide compositions and 12 additional hydrophilicity/hydrophobicity components would be incorporated in a feature vector fed into the predictor. Nevertheless, to evade from noise and overfitting problems, ANOVA was employed to further optimize the feature set. By calculating the* F*-value of each candidate feature and ranking them in descending order, a series of feature sets in various sizes were obtained based on IFS strategy as illustrated in* Feature Selection section*. The IFS curve reached its peak when the feature number was 374, indicating that the feature subset was the least redundant and the most discriminative one. Consequently, the AUC was improved from 0.85 to 0.93 and the computational time and expenses were acutely reduced.

### 3.2. Comparison with Other Classifiers

Subsequent to the determination of optimum parameters and feature subset, we further compared the performance between various classifiers, namely, SVM, Bayes Net, Radial Basis Function Network (RBF Network), and Random Forest, by applying the same sequence and feature data under the same parameters. The software WEKA (version 3.8) (http://www.cs.waikato.ac.nz/~ml/weka/downloading.html) was used to implement the last three classifiers. Likewise, calculations were made in line with the four metrics illustrated in* Performance Evaluation Section*. The results are summarized in Table 1. From Table 1, although relative similar Sns were produced by SVM (94.29%), Bayes Net (82.86%), and RBF Network (85.71%), the differences in other three metrics among the four classifiers were conspicuous enough to pronounce judgment. Nevertheless, Sn and Sp may lose objectivity because of an imbalance in class sizes, and even when all samples were misjudged as negative, the Sp and overall accuracy (OA) would still hang in high levels. Therefore, we attached more importance to Ac instead of Sn or Sp. As we can see from Table 1, SVM also achieved the maximum Ac with 87.18%, followed by Bayes Net (69.25%) and Random Forest (65.15%).

**Figure 1:**
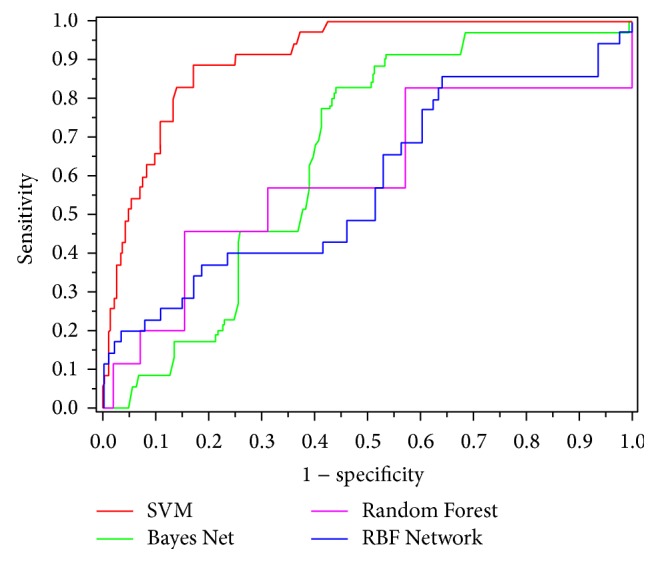
ROC curves achieved by SVM, Bayes Net, RBF Network, and Random Forest in discriminating secretory proteins from nonsecretory proteins of* M. tuberculosis*.

In fact, all the metrics mentioned above are static assessment indicators, except AUC, which emerges as a dynamic measure depending on ROC and remains objective under class imbalance conditions. Thus, AUC was chosen as the foremost evaluation criteria to compare performance disparities among the four algorithms. To visually view the dynamic changing process of Sn (also known as true positive rates or TPR) of a certain algorithm versus its Sp (also known as true negative rates or TNR), four curves corresponding to four classifiers were plotted in Figure 1 on the basis of their confusion matrixes varying with a range of thresholds. As shown in Figure 1, the SVM gave an AUC of 0.93 in the discrimination between secretory proteins and nonsecretory proteins, which is higher than that of the other three classifiers. Furthermore, the tendency of SVM curve was steeper and ran much closer to the vertical axis than that of the others, which signified a better performance in ROC curve. Thus, compared with the other three state-of-the-art classifiers, SVM is much more powerful and robust for classifying secretory and nonsecretory proteins. Two main reasons may lead to the result. SVM is insensitive to the number and probability distribution of training samples, along with the dimension of input space. Furthermore, SVM can gain the global optimal solution of goal function on the basis of convex optimization theory, while other classifiers based on greedy learning strategies were usually trapped into locally optimal solutions.

### 3.3. Web Server Construction

For the convenience of a vast majority of experimental scientists, a user-friendly web server, namely,* MycoSec*, was established, which is freely accessible at http://lin.uestc.edu.cn/server/MycoSec/. Below is a step-by-step guide on how to use the web server.

**Figure 2:**
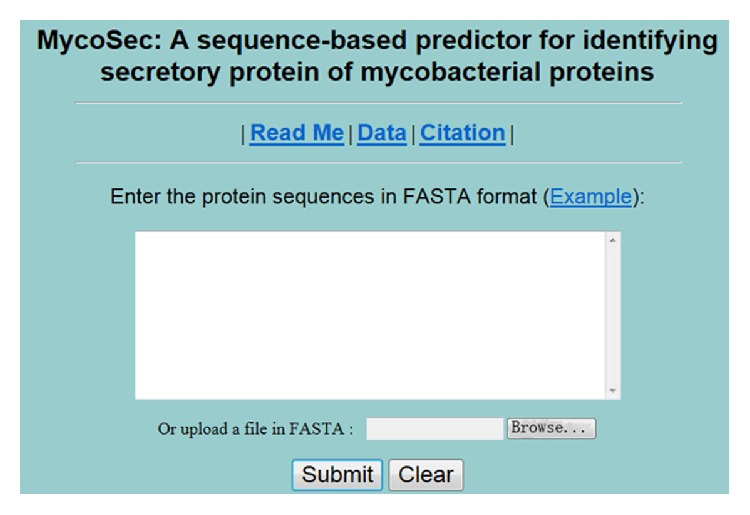
Home page of MycoSec web server at http://lin.uestc.edu.cn/server/MycoSec/.

First, reasonable and clear as you can see in Figure 2, the homepage provides several buttons and a textbox lying to the center of the whole. A brief introduction of the predictor and the caveat when using it would be present on clicking on the* Read Me* button.

Second, either type/paste the query protein sequences into the input box or select a FASTA file from a certain directory, ensuring that each sequence is longer than 11 amino acid residues and without any ambiguous characters. Example sequences in FASTA format can be seen by clicking on the* Example* button right above the input box.

Third, click on the* Submit* button to see the predicted results present in a new webpage. For a query sequence, the first column of the results shows its actual label, the second gives the probability that it belongs to secretory protein class, and the third is the probability that it pertains to nonsecretory protein class.

Fourth, the benchmark data used in our analysis can be downloaded by clicking on the* Data* button for training and testing our system or your own model.

Finally, click on the* Citation* button to find the relevant papers that document the detailed development and algorithm of* MycoSec*.

## 4. Conclusions

Identification of secretory proteins in* M. tuberculosis* is of paramount significance for targeting of antigens and vaccine development, which may contribute to an early diagnose or cure of the terrible disease tuberculosis. In this paper, we proposed a SVM-based algorithm to identify secretory proteins in* M. tuberculosis*. In this method, an improved pseudo amino acid composition which integrates *g*-gap dipeptides along with physicochemical properties was proposed and used to encode the residue sequences. By parameters search and feature optimization, we obtained the optimal model with an AUC of 0.93 validated by jackknife test. Based on this model, an online predictor* MycoSec* was established for the convenience of researchers in relevant fields. We hope the predictor will be a smart tool in* M. tuberculosis* research. In the future, we will combine other features such as evolution information to improve classification performance.

## Figures and Tables

**Table 1:** Comparing the performance between different classifiers.

Algorithm	Sn (%)	Sp (%)	AA (%)	AUC
SVM	94.29	80.08	87.18	0.93
Random Forest	45.71	84.59	65.15	0.69
Bayes Net	82.86	55.64	69.25	0.66
RBF Network	85.71	36.09	60.90	0.59
